# Quadruped Robot Control: An Approach Using Body Planar Motion Control, Legs Impedance Control and Bézier Curves

**DOI:** 10.3390/s24123825

**Published:** 2024-06-13

**Authors:** Gabriel Duarte Gonçalves Pedro, Gabriel Bermudez, Vivian Suzano Medeiros, Hélio Jacinto da Cruz Neto, Luiz Guilherme Dias de Barros, Gustavo Pessin, Marcelo Becker, Gustavo Medeiros Freitas, Thiago Boaventura

**Affiliations:** 1 Mechanical Engineering Department, São Carlos School of Engineering, University of São Paulo, São Carlos 13566-590, SP, Brazil; viviansuzano@usp.br (V.S.M.); helio.neto@usp.br (H.J.d.C.N.); luiz.barros@itv.org (L.G.D.d.B.); becker@sc.usp.br (M.B.); tboaventura@usp.br (T.B.); 2 Robotics Lab, Instituto Tecnologico Vale, Ouro Preto 35400-000, MG, Brazil; gustavo.pessin@itv.org; 3 Electrical Engineering Department, Federal University of Minas Gerais, Belo Horizonte 31270-901, MG, Brazil; gustavomfreitas@ufmg.br

**Keywords:** quadruped robots, Bézier curves, impedance control, legged control, body control

## Abstract

In robotics, the ability of quadruped robots to perform tasks in industrial, mining, and disaster environments has already been demonstrated. To ensure the safe execution of tasks by the robot, meticulous planning of its foot placements and precise leg control are crucial. Traditional motion planning and control methods for quadruped robots often rely on complex models of both the robot itself and its surrounding environment. Establishing these models can be challenging due to their nonlinear nature, often entailing significant computational resources. However, a more simplified approach exists that focuses on the kinematic model of the robot’s floating base for motion planning. This streamlined method is easier to implement but also adaptable to simpler hardware configurations. Moreover, integrating impedance control into the leg movements proves advantageous, particularly when traversing uneven terrain. This article presents a novel approach in which a quadruped robot employs impedance control for each leg. It utilizes sixth-degree Bézier curves to generate reference trajectories derived from leg velocities within a planar kinematic model for body control. This scheme effectively guides the robot along predefined paths. The proposed control strategy is implemented using the Robot Operating System (ROS) and is validated through simulations and physical experiments on the Go1 robot. The results of these tests demonstrate the effectiveness of the control strategy, enabling the robot to track reference trajectories while showing stable walking and trotting gaits.

## 1. Introduction

Recent advances have led to several commercial robotic platforms designed and sold for use in a variety of environments, focusing on the development of legged locomotion systems. Companies such as Boston Dynamics, ANYbotics, and Unitree are currently presenting commercial versions of quadruped robots for industry, mines, oil rigs, and other service applications [1,2,3].

Researchers have been exploring legged locomotion as a capability for mobile robots to navigate and accomplish tasks in unstructured and unsafe terrains [1,2,3,4]. Researchers have primarily focused on developing motion planning and control frameworks to improve safe locomotion for legged robots across diverse scenarios and environments in this domain. Examples of applications include autonomous mapping of large physical structures using quadruped robots [3], autonomous inspection of deterioration of concrete floors in sewer canals [2], litter removal from city coasts using a vacuum cleaner attached to one of the robot legs [5] and the study of quadruped robots for future planetary exploration missions [6].

To control the motion and balance of legged robots, nonlinear control techniques are often necessary due to the inherent nonlinearities in these systems. To enhance tractability, the feedback linearization technique is usually employed, both for control of the legs and the full robot. Typically, in robots featuring hydraulic and electric actuation, this involves calculating a torque that cancels nonlinear components and subsequently tracking this computed torque through low-level torque controllers for each joint [4,7,8,9]. Impedance control is also used in robots with hydraulic or electric actuators [10,11] and aims to adjust the stiffness and damping of the legs depending on the scenario. This change allows for absorbing external forces, due to terrain irregularity [11], and impact forces, as a result of dynamic steps [7], avoiding damage to the legs and actuators of the robot. Impedance control has been used in [12] to enable blind locomotion in rough terrain. The authors present an optimization-based approach to modulate the leg impedance to adapt to external disturbances and terrain variations. Similarly, ref. [13] also employed an optimization of impedance parameters but with the addition of a decomposition of the controller into force–position conversion factors and impedance subsystems, especially to improve the time response.

As noted in [14], the ability of animals to change their gait pattern is crucial to moving efficiently at different speeds and dealing with disturbances caused by irregularities in the ground. To achieve this mobility, a dedicated controller for each robot leg can be used to achieve the correct movement of each step based on the trajectory defined by a gait pattern. A common approach to deciding the position of the robot’s next step is to use Raibert’s heuristic [15], which calculates the robot’s next step position in the inertial reference frame based on the position of the body. This heuristic is widely used in different works with modifications depending on the application [16,17]. Another approach is to generate the steps and choose the positions of the feet in the robot’s reference frame with a cyclical movement of the feet. These trajectories must be smooth and adjustable based on the new motion and gait parameters of robots. A viable solution is to use Bézier curves, as shown in [18,19,20]. Another popular approach for control and trajectory planning for legged robots is the model predictive control (MPC) [17,21,22,23,24]. It can be employed with [17,21,22] or without [24] perceptive information and allows for terrain adaptation and disturbance rejection due to its predictive planning. However, the formulation can be quite complex for simpler tasks, and it usually involves tuning for the cost parameters. Computational cost can also be an issue depending on the dynamic model used for the optimization [23]. Finally, MPC can also be combined with reinforcement learning [21].

Other approaches with increasing popularity in robotics employ machine learning methods. These approaches explore the improvement of algorithms through the use of training data without the need for explicit programming [25]. This process facilitates the automation of analytical models for data analysis, decision-making and control, enabling robots to acquire new skills, adapt to changes in their environment, and improve their performance through experience rather than relying solely on pre-programmed instructions.

In quadruped robot control, one of the applications of machine learning is in decision-making for the optimal choice of foot support points. This concept is exemplified by [26], who employed a deep learning technique called convolutional neural networks. These neural networks were trained to learn and send to the robot control the relationship between the local elevation map and the quality of the potential support points, taking into account kinematic constraints, collisions and the geometric characteristics of each cell in the elevation map.

The reinforcement learning technique has emerged as an alternative for controlling the robot’s body, as seen in [27]. The technique employs reward functions and proprioceptive sensors to control joint and base positions, with a convolutional neural network trained in a simulated environment based on the robot’s model. In the simulation, the robot is trained in different procedurally generated environments, which are created with random procedures to ensure variability. Once the artificial neural networks had been trained, the control developed with the neural network in a simulated environment was applied to the ANYmal robot and tested in a variety of natural environments. These included steep mountain trails, streams with running water, mud, dense vegetation, loose gravel, snow-covered hills and a damp forest.

In a subsequent paper [28], the authors of [27] improved their study using a second neural network trained to move the ANYmal robot using the elevation maps generated using the exteroceptive sensor. The same approach was applied to both virtual training and real-world testing. The trained neural networks enabled more robust locomotion in challenging environments, even in situations where the data from the exteroceptive sensors is uncertain, including operation in environments with the presence of smoke, dust, translucent objects and lighting variations. In these challenging environments, the neural network relied more on information from proprioceptive sensors.

Another application of machine learning involves training a neural network to control the locomotion of various four-legged robotic platforms with related morphology, regardless of their dimensions and mass [29]. The training of this neural network used different models of quadruped robots with similar morphology, changing the lengths and masses of the links and bodies of the robots. This approach enabled the neural network to provide generalized locomotion control for various real quadruped robots without the need for explicit training on each specific robot.

The transfer of policies learned through machine learning methods represents a promising strategy for developing new robot skills. However, simulation approaches applied to real robots typically require manual design and tuning of reward parameters in addition to a simulation that represents reality well. In [30], a large language model is used to automate and accelerate the design of simulations that are both realistic and feasible. The large language model’s guided sim-to-real approach requires only the physics simulation for the target task and automatically constructs suitable reward functions and domain randomization distributions to support real-world transfer.

Machine learning methods hold significant promise for application in quadruped robots, as evidenced by numerous studies mentioned previously. Nonetheless, the high computational cost, challenges in obtaining accurate data, and risks of overfitting remain substantial hurdles [31]. These issues underscore the continued relevance and utility of model-based techniques in this field.

This paper is based on the previously presented conference papers [32,33,34] and is an extension of the paper [34]. The main extensions include (1) an experimental test for validation of the results with a real quadruped robot; and (2) a deeper explanation of the planar control of the base.

This paper employs a methodology akin to that presented in [20], with notable distinctions delineated subsequently. The work [20] considers a quadruped robot with legs modeled using Euler–Lagrange formalism and applies impedance control to the legs. Specifically, an eleven-degree Bezier curve featuring twelve control points governs leg trajectories during the swing phase, while a sinusoidal wave characterized by step length and amplitude parameters dictates the penetration depth of step trajectories into the ground, thereby generating impulsive forces. The two curves are in 2D space. The gait generator is based on the desired body speed, which determines the period of the foot support phase. It modulates the steps and gait pattern according to the speed.

The current paper also considers quadruped-legged robots modeled using Euler–Lagrange equations. The control strategy adopts feedback linearization combined with impedance control for each leg. The reference trajectories for each leg are based on a single curve of sixth-degree Bézier curves with seven control points for the step support and swing phases in 3D space. The steps are generated according to the desired leg velocities, which modulate the step frequency and length. The modulated step frequency determines the duration of the support phase using a foot occupancy factor, which was kept constant at 0.8. The steps were synchronized to emulate static walking and trotting gaits, without changing the gait pattern as a function of speed.

The speed of each leg is obtained from a body controller based on a planar kinematic model. This approach using kinematic and dynamic modeling is similar to the one used in [35] that proposes a kinematic control of the center of mass position augmented with a dynamic feedback zero-moment point regulator to improve the dynamic stability of a biped robot. The outcome of this consideration is a responsive scheme to external disturbances, achieved through dynamic feedback used in conjunction with kinematic whole-body control. This is accomplished without requiring a precise model of the robot and environment, which can be highly nonlinear and difficult to obtain [35]. In the current paper, a planar differential kinematic model of the robot’s body is employed to correlate the desired velocities of the body with the desired velocities of the robot’s legs, which are sent to a dynamic impedance controller using only the model of the robot’s legs.

In implementing the control strategy, the Locosim robotics framework [36] serves as the platform of choice. It encompasses a Python ROS Noetic node for high-level planning and control, interfacing with a C++ ROS Noetic control node for low-level control to a Gazebo v11 simulator or physical hardware. The Unitree Go1 quadruped robot platform is employed for both simulations and real hardware experiments. The Unitree Go1 robot description is already integrated into the Locosim framework for both simulation and real hardware. For the body and leg control strategies, we developed a custom high-level Python ROS Noetic node and a low-level C++ ROS Noetic node based on Locosim. The high-level node uses Pinocchio [37] to compute the kinematics and dynamics of the robot, and the low-level node sends the commands to the robot through the hardware interface. The proposed approach is validated in simulations and experiments with the Unitree Go1 robot, which shows that the robot is capable of following a reference curve such as the Bernoulli lemniscate, employing an impedance control for the legs following the chosen gait pattern.

To summarize, the main contributions of this paper are:

- A planar kinematic model of the robot body inspired by differential robots;
- Step trajectory planner using a single Bézier curve for the swing and support phases of feet without decomposition of the walking cycle;
- Algorithm for calculating step frequency and length based on the step speed of the robot’s legs.
- The validation of the proposed control conducted through simulations and experiments with a physical robot.

## 2. Materials and Methods

This section describes the modeling and control methodologies employed in this article. It includes the dynamic modeling and control of the legs, the generation of steps and gait patterns using Bézier curves, the development of a planar kinematic model of the body, and a general description of the control strategy.

### 2.1. Dynamic Model and Control of the Legs

The dynamics of a three Degrees of Freedom (DoF) quadruped robot’s leg derived using Euler–Lagrange equations are [38]:

(1) $$M\left(q\right)\;\ddot{q}+C\left(q,\dot{q}\right)\;\dot{q}+g\left(q\right)+J^{T}\left(q\right)\;F_{e}=\tau ,$$

where $M\left(q\right)\in R^{3\times 3}$ is the inertia matrix, $C\left(q,\dot{q}\right)\in R^{3\times 3}$ is the Coriolis force matrix, $g\left(q\right)\in R^{3}$ is the vector of gravitational forces, $\tau \in R^{3}$ is the vector of joint torques, *q*, $\dot{q}$ and $\ddot{q}\in R^{3}$ are the positions, velocities and accelerations of the three joints, respectively. The resulting torques of external forces are calculated with a transpose of the Jacobian of the foot $J^{T}\left(q\right)\in R^{3\times 3}$ and the vector of external forces $F_{e}={\left[F_{x},F_{y},F_{z}\right]}^{T}$.

We propose to use the following inverse dynamics control law, where $a_{q}\in R^{3}$ and $a_{f}\in R^{3}$ are auxiliary control inputs of acceleration and force, respectively:

(2) $$\tau =M\left(q\right)\;a_{q}+C\left(q,\dot{q}\right)\;\dot{q}+g\left(q\right)+J^{T}\left(q\right)\;a_{f}.$$

To map Equation (2) to the foot task-space, we use the definition of Jacobian, that is:

(3) $$\dot{q}=J{\left(q\right)}^{-1}\dot{x},$$

(4) $$\ddot{q}=J{\left(q\right)}^{-1}\left[\ddot{x}-\dot{J}\left(q,\dot{q}\right)\dot{q}\right],$$

where $\dot{x}$ and $\ddot{x}\in R^{3}$ are the velocity and linear acceleration vectors in the foot task-space. Changing the variables $\ddot{q}$ and $\ddot{x}$ from Equation (4) to $a_{q}$ and $a_{x}$, substituting them into Equation (2), and entering the result into Equation (1) yields Equation (5).

(5) $$\ddot{x}=a_{x}+J\left(q\right)M^{-1}\left(q\right)J^{T}\left(q\right)\left(F_{e}-a_{f}\right).$$

For simplification purposes, it is assumed that $a_{f}=F_{e}$ to recover the double integrator system from the task-space [38]. With this simplification and assuming that any additional force feedback terms are included in the control term, $a_{x}$ is obtained using Equation (6).

(6) $$\ddot{x}=a_{x}.$$

The idea of impedance control is to regulate mechanical impedance, i.e., apparent inertia, damping, and stiffness, by force feedback [38]. For the auxiliary control $a_{x}$, the following impedance control law is proposed:

(7) $$a_{x}={\ddot{x}}_{d}-M_{x_{d}}^{-1}\;\left[B_{x_{d}}\;\left(\dot{x}-\dot{x_{d}}\right)+K_{x_{d}}\;\left(x-x_{d}\right)+\;F_{e}\right],$$

where the subscript *d* indicates desired values. Substituting Equation (7) into Equation (6) and defining the error dynamics as $e=(x-x_{d})$, we finally obtain:

(8) $$M_{x_{d}}\;\ddot{e}+B_{x_{d}}\;\dot{e}+K_{x_{d}}\;e=\;-F_{e}.$$

The final control torque can be seen as a composition of two components:

(9) $$\tau =\tau_{ff}+\tau_{fb},$$

where:

(10) $$\tau_{ff}=M\left(q\right){J(q)}^{-1}\left({\ddot{x}}_{d}-\dot{J}\left(q,\dot{q}\right)\dot{q}-{M_{x_{d}}}^{-1}F_{e}\right)+C\left(q,\dot{q}\right)\;\dot{q}+g\left(q\right)+J^{T}\left(q\right)F_{e},$$

represents the feed-forward inverse dynamics torque,

(11) $$\tau_{fb}=-M\left(q\right){J(q)}^{-1}{M_{x_{d}}}^{-1}\left(K_{x_{d}}e+B_{x_{d}}\dot{e}\right),$$

which represents the feedback torque.

### 2.2. Bézier Curves

The basic mathematics for the development of Bézier curves was established in 1912, but these polynomials were not widely disseminated until the 1960s by French engineer Pierre Bézier [39]. In robotics, Bézier curves can be used to plan trajectories for manipulators and mobile robots [40,41,42,43,44,45,46].

The Bézier curve B(u) is defined as the curve formed by the linear interpolation of a set of weights $W_{0}$ to $W_{n+1}$, where n∈N corresponds to its degree. This curve is constructed using Equation (12), which defines a Bézier curve of any order as:

(12) $$B\left(u\right)=\sum_{i=0}^{n}\left(\begin{array}{c} n\\ i \end{array}\right){\left(1-u\right)}^{n-i}\;u^{i}\;W_{i},$$

where $\left(\begin{array}{c} n\\ i \end{array}\right)$ is the binominal coefficient calculated as:

(13) $$\left(\begin{array}{c} n\\ i \end{array}\right)=\frac{n!}{i!\;(n-i)!}.\;$$

The term $B\left(u\right)\in R^{m}$ represents the Bézier curve, $W_{i}\in R^{m}$ are weight vectors of the curve, *u* is the parameterization variable of the curve where $\left\{\begin{array}{c} u\in R\;|\;0\leq u\leq 1 \end{array}\right\}$. The Bézier curve can have a desired travel time by correlating the parameterization interval *u* from 0 to 1 with time. The Bézier curve can even correlate different travel times in segments of the Bézier curve, as long as it respects the parameterization interval 0 to 1. The derivatives of a Bézier curve are also reduced-degree Bézier curves and can be obtained recursively with Equation (14):

(14) $$\begin{array}{c} \dot{B}\left(u\right)=\sum_{i=0}^{k}\left(\begin{array}{c} k\\ i \end{array}\right){\left(1-k\right)}^{k-i}\;u^{i}\;n\;\left(W_{i+1}-W_{i}\right), \end{array}$$

where $\left(\begin{array}{c} k\\ i \end{array}\right)$ corresponds to the binomial coefficient of the derivative, *k* is the reduced degree of the Bézier curve $\left(k=n-1\right)$, and $n\;(W_{i+1}-W_{i})$ is the calculation of the new weight vectors based on the degree and control points of the derived Bézier curve.

#### Curve Fitting

The weights $W_{i}$ defined previously can be adjusted such that the error between the actual curve and a set of control points $P_{i}$, including the parameterization interval *u* at which each one of these control points is reached, is minimized. The evaluation of these weights using a standard least squares fitting procedure gives Equation (15):

(15) $$\begin{array}{c} W=M^{-1}\;{\left(T^{T}\;T\right)}^{-1}\;T^{T}\;P, \end{array}$$

where $W\in R^{(n+1)\times m}$ is the matrix referring to the weights, $W_{i}$ is each corresponding row, $M\in R^{\left(n+1)\times (n+1\right)}$ is the diagonal matrix of the Bézier curve referring to the binomial coefficients obtained by polynomial expansion of the Bézier curve, $P\in R^{(n+1)\times m}$ corresponds to the matrix of curve fitting points, where each row of the matrix refers to $P_{i}$, and $T\in R^{\left(n+1)\times (n+1\right)}$ is a matrix with temporal information, whose rows are vectors $u_{i}=\left[1\;u\;u^{2}\;\ldots \;u^{n}\right]$ containing the times determined for $P_{i}$ within the range 0 to 1.

### 2.3. Bézier Curves for Steps

For the robot to walk, the feet must move by taking steps. A step can be divided into a support phase and a swing phase, repeating this cycle in different gait patterns. In order to define a smooth curve using the foot trajectories for impedance control, Bézier curves were employed, such as [18,19].

We use a single sixth-degree Bézier curve, previously proposed in [33], traversing a closed trajectory for both phases of the step. This closed curve is defined by seven control points, with the aim of simplifying the planning of the swing and foot support trajectories in a reduced solution space. We chose a sixth-degree curve that could generate a suitable trajectory with a support time greater than the foot-switch time in the parametrization range for the robot’s foot motion.

The equation defining a Bézier curve $B\left(u\right)\in R^{3}$ can be rewritten in matrix form using the polynomial expansion given in Equation (16). The weight vectors of the curve and the parameterization variable are represented by $W_{i}\in R^{3}$ and *u*, where $\left\{\begin{array}{c} u\in R\;|\;0\leq u\leq 1 \end{array}\right\}$. The derivatives of a Bézier curve can be obtained recursively with the characteristic equation of its derivatives. The Bézier curve fitting was set at the control points $P_{i}$ and the parameterization range value within the specified range *u* in order to calculate the weights of the Bézier curve $W_{i}$ using Equation (15). For the 6th-degree Bézier curve employed, $W\in R^{7\times 3}$ is the weight matrix, $M\in R^{7\times 7}$ is the diagonal matrix of the binomial coefficients of the curve, $P\in R^{7\times 3}$ are the curve fit points, and $T\in R^{7\times 7}$ is a matrix with temporal information. Each row of the matrices *W* and *P* corresponds to a weight and a setpoint, respectively, while the rows of matrix *T* are vectors $u_{i}=\left[1\;u\;u^{2}\;\ldots \;u^{6}\right]$ with the times determined for each setpoint $P_{i}$ within the range 0 to 1:

(16) $$\begin{array}{c} \;\;\;\;B\left(u\right)=\;\;{\left[\begin{array}{c} 1\\ u\\ u^{2}\\ u^{3}\\ u^{4}\\ u^{5}\\ u^{6} \end{array}\right]}^{T}\left[\begin{array}{ccccccc} \;\;\;1 & \;\;\;0 & \;\;\;0 & \;\;\;0 & \;\;\;0 & \;\;\;0 & \;0\\ -6 & \;\;\;6 & \;\;\;0 & \;\;\;0 & \;\;\;0 & \;\;\;0 & \;0\\ \;\;\;15 & -30 & \;\;\;15 & \;\;\;0 & \;\;\;0 & \;\;\;0 & \;0\\ -20 & \;\;\;60 & -60 & \;\;\;20 & \;\;\;0 & \;\;\;0 & \;0\\ \;\;\;15 & -60 & \;\;\;90 & -60 & \;\;\;15 & \;\;\;0 & \;0\\ -6 & \;\;\;30 & -60 & \;\;\;60 & -30 & \;\;\;6 & \;0\\ \;\;\;1 & -6 & \;\;\;15 & \;-20 & \;\;\;15 & -6 & \;1 \end{array}\right]\;\;\left[\begin{array}{c} W_{0}\\ W_{1}\\ W_{2}\\ W_{3}\\ W_{4}\\ W_{5}\\ W_{6} \end{array}\right]. \end{array}$$

By recursively applying Equation (14), we derive the first-order derivative $\dot{B}\left(u\right)\in R^{3}$, as described in Equation (17), and subsequently, the second-order derivative $\ddot{B}\left(u\right)\in R^{3}$, as outlined in Equation (18), both presented in matrix form:

(17) $$\begin{array}{c} \dot{B}\left(u\right)={\left[\begin{array}{c} 1\\ u\\ u^{2}\\ u^{3}\\ u^{4}\\ u^{5} \end{array}\right]}^{T}\left[\begin{array}{cccccc} \;\;\;1 & \;\;0 & \;\;0 & \;\;0 & \;\;0 & \;\;0\\ -5 & \;\;5 & \;\;0 & \;\;0 & \;\;0 & \;\;0\\ \;\;\;10 & -20 & \;\;10 & \;\;0 & \;\;0 & \;\;0\\ -10 & \;\;\;30 & -30 & \;\;10 & \;\;0 & \;\;0\\ \;\;5 & -20 & \;\;\;30 & -20 & \;\;5 & \;\;0\\ -1 & \;\;\;5 & -10 & \;\;10 & -5 & \;\;1 \end{array}\right]\left[\begin{array}{c} W_{0}^{\prime }\\ W_{1}^{\prime }\\ W_{2}^{\prime }\\ W_{3}^{\prime }\\ W_{4}^{\prime }\\ W_{5}^{\prime } \end{array}\right], \end{array}$$

where $W_{i}^{\prime }=6\left(W_{i+1}-W_{i}\right)$ corresponds to the calculation of the weights of $\dot{B}\left(u\right)$,

(18) $$\begin{array}{c} \ddot{B}\left(u\right)={\left[\begin{array}{c} 1\\ u\\ u^{2}\\ u^{3}\\ u^{4} \end{array}\right]}^{T}\left[\begin{array}{cccccc} \;\;\;1 & \;\;\;0 & \;\;\;0 & \;\;0 & \;\;0 & \\ -4 & \;\;\;4 & \;\;\;0 & \;\;0 & \;\;0 & \\ \;\;\;6 & -12 & \;\;\;6 & \;\;0 & \;\;0 & \\ -4 & \;\;\;12 & -12 & \;\;4 & \;\;0 & \\ \;\;1 & \;-4 & \;\;\;6 & -4 & \;\;1 \end{array}\right]\left[\begin{array}{c} W_{0}^{\prime \prime }\\ W_{1}^{\prime \prime }\\ W_{2}^{\prime \prime }\\ W_{3}^{\prime \prime }\\ W_{4}^{\prime \prime } \end{array}\right], \end{array}$$

and $W_{i}^{\prime \prime }=5\left(W_{i+1}^{\prime }-W_{i}^{\prime }\right)$ indicates the calculation of the $\ddot{B}\left(u\right)$ weights.

### 2.4. Step and Gait Pattern Using Bézier Curves

The gait pattern defines how a legged robot moves. In the study of animal gait patterns presented in [47], some terms were defined to characterize a step and the gait pattern, including step, step frequency, step length, occupancy factor and relative phase.

**Figure 1:**
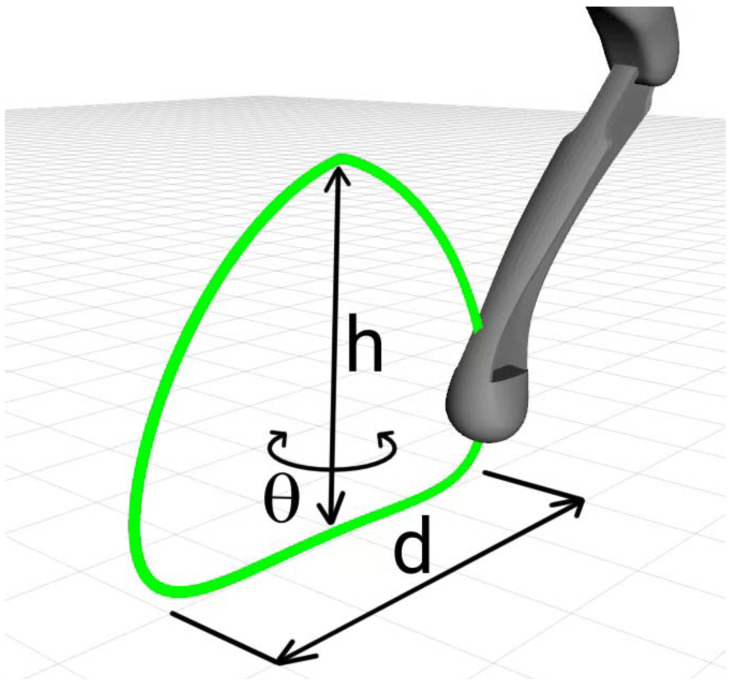
Bézier curve (green) to implement the support and swing phases during the step of a leg visualized in 3D with the *d*, *h* and θ parameters.

Based on these terms, we defined the starting and ending points ($P_{0}$ and $P_{6}$) of the Bézier curve that must coincide to form a closed trajectory that represents a step cycle. The beginning and end of the motion occur halfway through the swing phase of the leg. The distance variables d∈R for the step length, height h∈R of the step and the angular variable θ∈S were established to determine the direction of the step in space. A visualization of these parameters is shown in Figure 1.

By setting the $P_{3}$ point of the step in the foot task-space, the other six curve fit points can be derived as:

(19) $$\begin{array}{ccc} d_{x} & = & d\;0.7\;cos\left(\theta \right),\;d_{y}=d\;0.7\;sin\left(\theta \right),\\ P_{0} & = & [P_{3x},P_{3y},P_{3z}+h],\\ P_{1} & = & [P_{3x}+d_{x}\;\left(4/5\right),P_{3y}+d_{y}\;\left(4/5\right),P_{3z}+h\;\left(3/5\right)],\\ P_{2} & = & [P_{3x}+d_{x}\;\left(5/5\right),P_{3y}+d_{y}\;\left(5/5\right),P_{3z}+h\;\left(1/5\right)],\\ P_{3} & = & [P_{3x},P_{3y},P_{3z}],\\ P_{4} & = & [P_{3x}-d_{x}\;\left(5/5\right),P_{3y}-d_{y}\;\left(5/5\right),P_{3z}+h\;\left(1/5\right)],\\ P_{5} & = & [P_{3x}-d_{x}\;\left(4/5\right),P_{3y}-d_{y}\;\left(4/5\right),P_{3z}+h\;\left(3/5\right)],\\ P_{6} & = & P_{0}. \end{array}$$

The $u_{i}$ time instants were set to obtain a duty factor $\beta_{u}$ of 0.6, ensuring a deformation-free trajectory. Once the points *P* and the corresponding times are determined, the weights *W* can be calculated using Equation (15) and thus the weights of the derivatives. After the evaluation of all these parameters, the corresponding Bézier curve for the leg step can be obtained.

**Figure 2:**
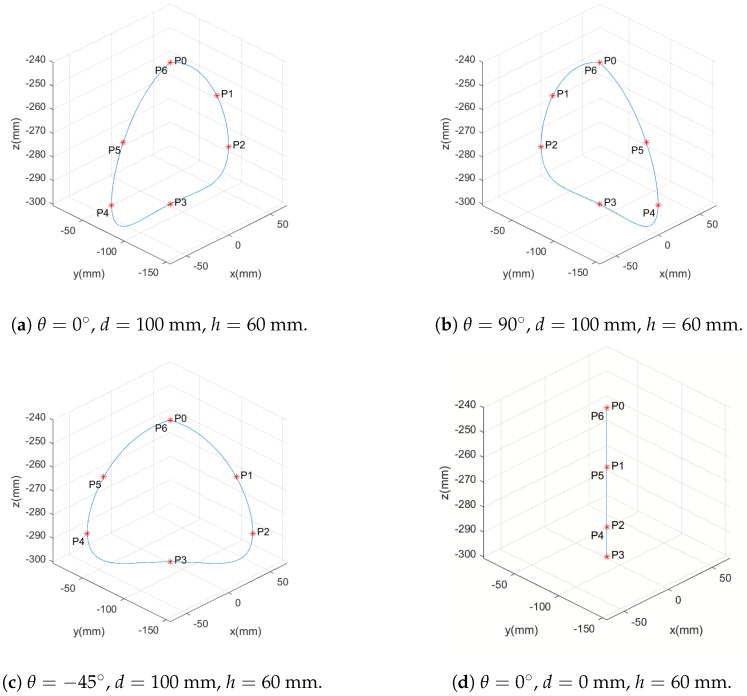
Bézier curves in space for different parameters with visualization of the fit of the *P* points.

To exemplify the calculated Bézier curves, Figure 2 illustrates steps in space at different orientations, with a step length of d=100 mm and d=0 mm, and height of h=60 mm. $P_{3}=\left[0,-0.085,-0.3\right]$ was chosen as the starting point of the steps, which is equivalent to the point below the right front shoulder of the robot.

Bézier curves represent a one-step cycle with a step frequency of 1 Hz and are parameterized by *u* in the range 0 to 1. It is possible to modify the step frequency by changing the map between *t* and *u*. The duty factor of the step, which is the ratio between the support phase and the total duration of each step, also changes with the mapping between *t* and *u*. We parameterize these phases of the Bézier curve with the desired duty factor $\beta_{t}$ for the step and the support phase between points $P_{2}$ to $P_{4}$ and the swing phase between points $P_{0}$ to $P_{2}$ and $P_{4}$ to $P_{6}$. The parameterized curve at *u* has a duty factor $\beta_{u}$ between the points $P_{2}$ to $P_{4}$ of 0.6. The parameterization of the variable *u* with respect to time *t* of a period $T_{p}=1/f_{p}$ and with a desired duty factor $\beta_{t}$ obtained by Equation (20):

(20) $$\begin{array}{c} u\left(t\right)=\left\{\begin{array}{c} \frac{t}{T_{p}}\;\left(\frac{\beta_{u}\;-1}{\beta_{t}-1}\right)\;,\;\;0⩽\frac{t}{T_{p}}<\frac{1-\beta_{t}}{2}\\ \frac{\beta_{u}\;\left(\frac{\left(2\;t\right)}{T_{p}}-1\right)}{2\;\beta_{t}}+\frac{1}{2}\;,\;\;\frac{1-\beta_{t}}{2}⩽\frac{t}{T_{p}}⩽\frac{\beta_{t}+1}{2}\\ \frac{\beta_{u}}{2}-\frac{\left(\beta_{u}-1\right)\;\left(\beta_{t}-\frac{\left(2\;t\right)}{T_{p}}+1\right)}{2\;(\beta_{t}-1)}+\frac{1}{2}\;,\;\;\frac{\beta_{t}+1}{2}<\frac{t}{T_{p}}⩽1 \end{array}\right.. \end{array}$$

The chosen gait patterns for reproduction were static walking and trot, akin to those presented in [48,49,50]. For symmetric patterns, the step length and the position of its start were designed to ensure a polygon or line of support for the center mass of the body [48,49].

**Figure 3:**
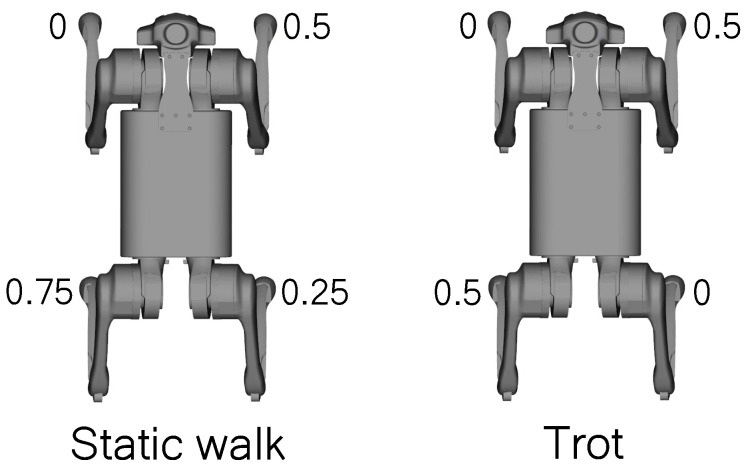
Diagrams of relative-phase for static walking and trotting.

**Figure 4:**
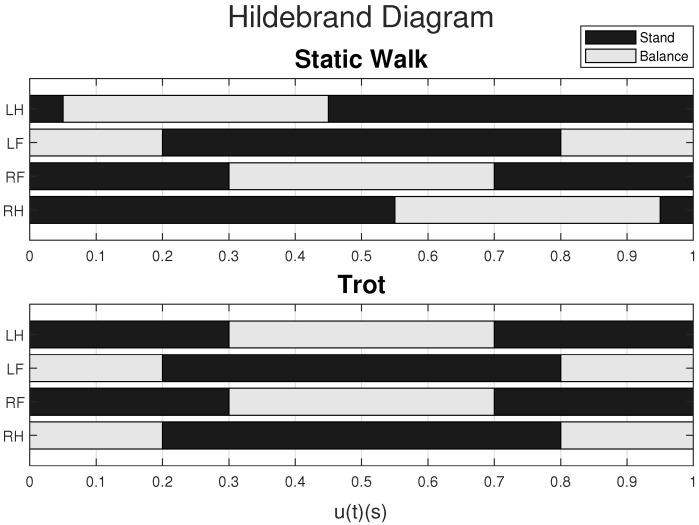
Hildebrand diagram with an occupancy factor of 0.8.

The robot gait pattern is defined by determining the relative phases of each leg for the locomotion pattern, with the aim of synchronizing the movement of the legs to maintain the support of the center of mass [51]. The relative step phases, defined for the Bézier curves of each leg for these gait patterns, are illustrated in the diagram in Figure 3 and can be visualized with the Hildebrand diagram [51] in Figure 4.

The average speed of locomotion with legs, $v_{mp}$, considering successful and constant steps, can be estimated by multiplying the frequency of the step $f_{p}$ by its length *d* [47], using Equation (21):

(21) $$\begin{array}{c} v_{mp}=d\;f_{p}. \end{array}$$

Using this equation, a function was developed to calculate the frequency and step length for a given leg speed in a step frequency range. This function is described in Algorithm 1.

**Figure 5:**
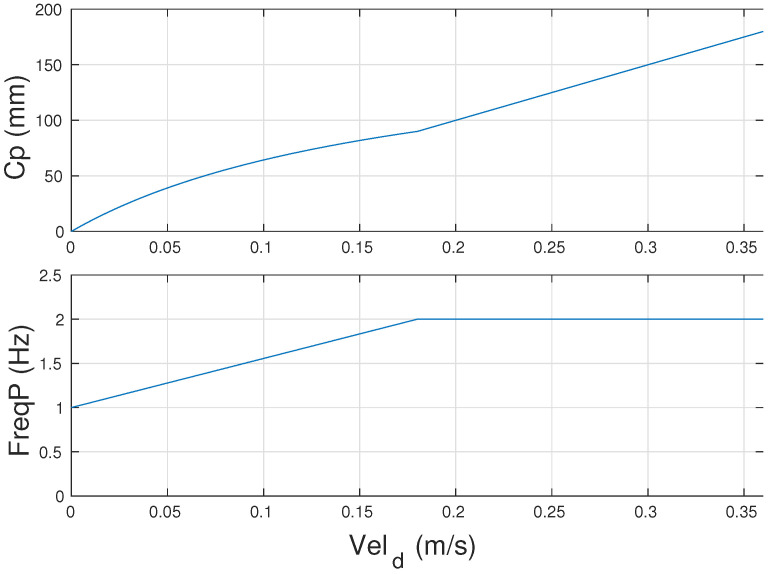
Result of the function for calculating step length ($C_{p}$) and frequency (FreqP) for speeds up to 0.36 m/s.

This function scans the maximum and minimum frequency range for different step lengths in the range from zero to the maximum step length until it finds a solution to Equation (21) for the desired speed. For this paper, the range of 1 to 2 steps per second was considered, with a maximum step length of 180 mm. In these conditions, the function *calc_cp_freqP* has the following results for speeds up to 0.36 m/s, as shown in Figure 5.
**Algorithm** **1:** Function for calculating frequency and step length.
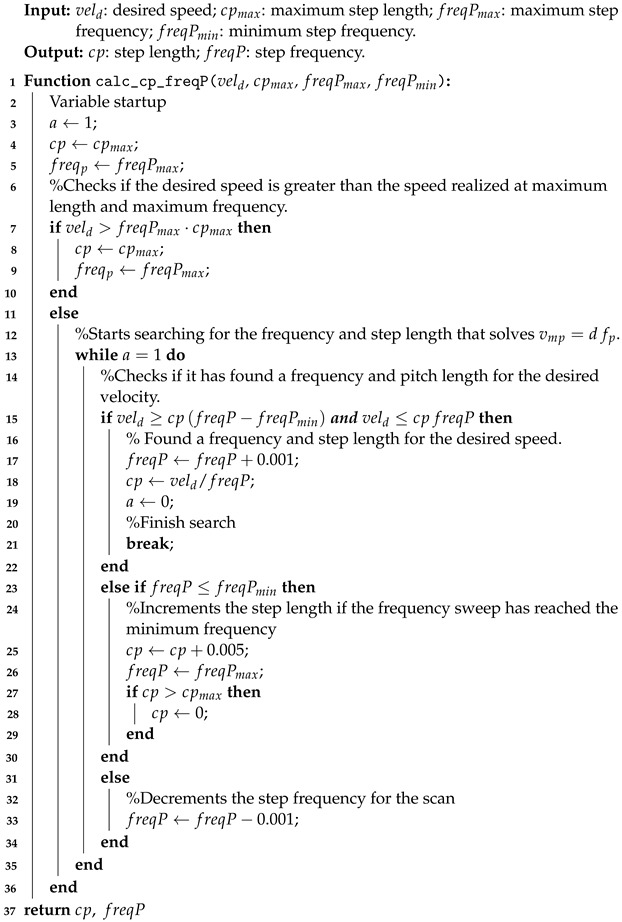


The *calc_cp_freqP* function makes the step frequency vary linearly with the speed until it reaches the maximum step frequency. It also makes it so that the maximum step length is only reached when it is close to the maximum speed, calculated by the maximum length and maximum frequency. The maximum length is chosen to stay within the working area of the robot’s feet.

This function is used to calculate the frequency and step length of the leg with the highest speed, and the step frequency calculated is used to determine the step length of the other legs with Equation (21). In this way, it is possible to modify the step frequency within this range as the desired speed increases, keeping the steps in sync because they are at the same frequency.

The relative phase was defined based on the chosen gait pattern, as illustrated in Figure 3. From these calculations and definitions for relative phase, height, direction and step length, the gait generator finds a 6th-degree Bézier curve and its derivatives for each leg, which are used to define the reference positions, velocities and accelerations of each foot in the workspace.

### 2.5. Planar Kinematic Model of the Body

**Figure 6:**
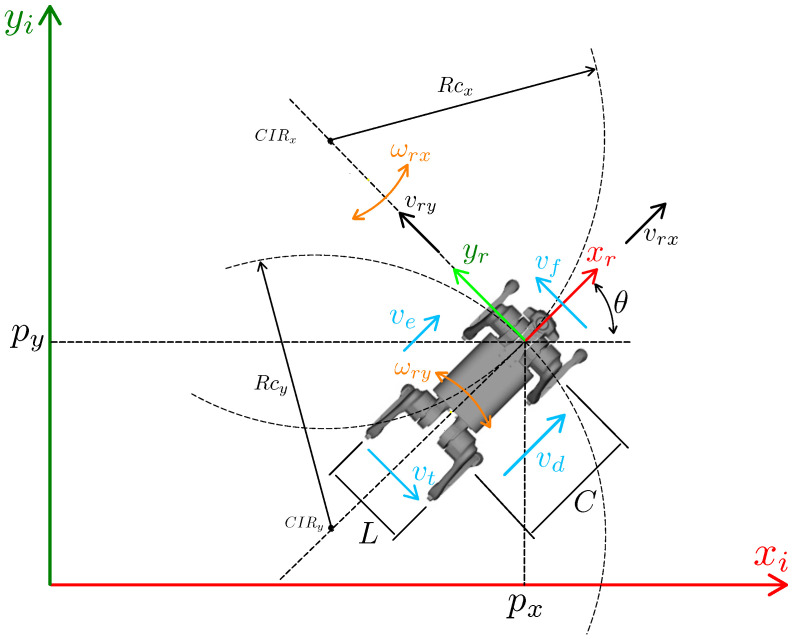
Quadruped robot planar diagram.

A differential kinematic model of a mobile robot with differential architecture is one of the simplest models used for wheeled robots. However, the differential kinematic model is inappropriate for quadruped robots because of the limitations of non-holonomic wheels. This paper proposes a simple planar kinematic model for quadruped robots, based on the differential model of a mobile robot, but including the lateral motion of the legs. This model assumes linear velocities in $x_{r}$ and $y_{r}$ directions in a plane, plus the angular velocity $\omega_{r}$ in *z*, and considers non-sliding feet during motion. Figure 6 illustrates a quadruped robot, the variables used, and the robot’s coordinate system, positioned at the center of a straight-line segment connecting the robot’s front shoulders in order to simplify the mathematical modeling and movement of the robot. The robot’s position will be determined by utilizing this coordinate system as a reference. Lateral velocities $v_{d}$, and $v_{e}$, front $v_{f}$ and rear $v_{t}$, width *L* and length *C* of the robot, as well as orientation θ in a plane, curvature radius $Rc_{x}$ and $Rc_{y}$ and instantaneous center of rotation $CIR_{x}$ and $CIR_{y}$ are represented in Figure 6.

Based on this diagram in Figure 6 and inspired by the differential robot model, we can obtain Equations (22) and (23) for the velocity $v_{e}$ and $v_{d}$ for the robot’s $x_{r}$ direction:

(22) $$\begin{array}{c} v_{e}=\omega_{rx}\left(Rc_{x}-\frac{L}{2}\right), \end{array}$$

(23) $$\begin{array}{c} v_{d}=\omega_{rx}\left(Rc_{x}+\frac{L}{2}\right). \end{array}$$

If we isolate the terms $\omega_{rx}\;Rc_{x}$ and then equate these terms, we can obtain the following relationship for the angular velocity $\omega_{rx}$:

(24) $$\begin{array}{c} \omega_{rx}=\frac{v_{d}-v_{e}}{L}. \end{array}$$

By performing the same variable isolation for $\omega_{rx}$ in Equations (22) and (23), it is possible to obtain an equation for the radius of curvature $Rc_{x}$:

(25) $$\begin{array}{c} Rc_{x}=\frac{L}{2}\frac{\left(v_{e}+v_{d}\right)}{\left(v_{d}-v_{e}\right)}. \end{array}$$

It is known that $v_{rx}=w_{rx}\;Rc_{x}$, and by substituting Equations (24) and (25) into this equation of $v_{rx}$, it is possible to obtain:

(26) $$\begin{array}{c} v_{rx}=\frac{v_{e}+v_{d}}{2}. \end{array}$$

For the $y_{r}$ direction, it is possible to obtain the following Equations (27) and (28) for $v_{f}$ and $v_{t}$:

(27) $$\begin{array}{c} v_{f}=\omega_{ry}\;Rc_{y}, \end{array}$$

(28) $$\begin{array}{c} v_{t}=\omega_{ry}\left(Rc_{y}-C\right). \end{array}$$

If we isolate the terms $\omega_{ry}\;Rc_{y}$ and equalize them, we can obtain the following relationship for the angular velocity $\omega_{ry}$:

(29) $$\begin{array}{c} \omega_{ry}=\frac{\left(v_{f}-v_{t}\right)}{C}. \end{array}$$

Performing the same for $\omega_{ry}$ with Equations (27) and (28), the Equation (30) for $Rc_{y}$ is found:

(30) $$\begin{array}{c} Rc_{y}=\frac{v_{f}\;C}{\left(v_{f}\;-v_{t}\right)}. \end{array}$$

Knowing that $v_{ry}=w_{ry}\;Rc_{y}$ and subsisting Equations (29) and (30) in this equation, it is possible to obtain the equation for $v_{ry}$:

(31) $$\begin{array}{c} v_{ry}=v_{f}. \end{array}$$

For angular velocity $\omega_{r}$, we can say that $\omega_{r}=\omega_{rx}=\omega_{ry}$ because they are velocities that refer to the same body. Based on this premise, it is possible to obtain the sum $2\;\omega_{r}=\omega_{rx}+\omega_{ry}$. Thus, by isolating $\omega_{r}$, we find the following relationship for the angular velocity of the body $\omega_{r}$:

(32) $$\begin{array}{c} \omega_{r}=\frac{v_{d}-v_{e}}{2\;L}+\frac{v_{f}-v_{t}}{2\;C}. \end{array}$$

By organizing Equations (26), (31), and (32) in matrix form, Equation (33) gives the direct differential kinematics of the robot, relating the velocities $v_{d}$, $v_{e}$, $v_{f}$ and $v_{t}$ to the velocities $v_{rx}$, $v_{ry}$ and $\omega_{r}$ in the robot’s referential:

(33) vrxvryωr=12120000101(2L)−1(2L)1(2C)−1(2C)vdvevfvt.

The inverse differential kinematics are found from the system formed by Equations (26), (24), (31) and (29) and knowing that $\omega_{r}=\omega_{rx}=\omega_{ry}$. By making the appropriate substitutions for the system presented, the following equations are found for the inverse differential kinematics:

(34) $$\begin{array}{ccc} v_{d} & = & v_{rx}+w_{rx}\;\frac{L}{2}, \end{array}$$

(35) $$\begin{array}{ccc} v_{e} & = & v_{rx}-w_{rx}\;\frac{L}{2}, \end{array}$$

(36) $$\begin{array}{ccc} v_{f} & = & v_{ry}, \end{array}$$

(37) $$\begin{array}{ccc} v_{t} & = & v_{ry}-w_{ry}\;C. \end{array}$$

Organizing these equations in matrix form gives Equation (38) for the inverse kinematics, correlating the robot’s velocities $v_{rx}$ and $v_{ry}$, and $w_{r}$ with the velocities $v_{d}$, $v_{e}$, $v_{f}$ and $v_{t}$:

(38) vdvevfvb=10L210−L201001−Cvrxvryωr.

Using the same approach as the schematic in Figure 6, but considering the robot’s coordinates at the center of the robot’s body, it is possible to find the direct and inverse differential kinematics, Equations (39) and (40), for the center of the body.

(39) vrxvryωr=1212000012121(2L)−1(2L)1(2C)−1(2C)vdvevfvt,

(40) vdvevfvb=10L210−L201C201−C2vrxvryωr.

To obtain the velocity of each leg of the robot, we use the modulus of the velocities of the vertices formed by the width and length of each leg. When the robot’s feet perform non-slip steps, the velocity of the foot motion is transferred to the shoulders. Equation (41) calculates the modulus of the velocities front right ($v_{fd}$), front left ($v_{fe}$), rear right ($v_{td}$) and rear left ($v_{te}$) legs. The angle indicating the direction of leg velocities with respect to the robot referential is calculated using the atan2 function of the velocities of the vertices of the width and length of the legs, using Equation (42). The obtained velocities and direction of each leg are then used to control the motion of the robot legs:

(41) $$\begin{array}{c} v_{fd}=\sqrt{{v_{f}}^{2}+{v_{d}}^{2}},\;\;v_{fe}=\sqrt{{v_{f}}^{2}+{v_{e}}^{2}},\;\;v_{td}=\sqrt{{v_{t}}^{2}+{v_{d}}^{2}},\;\;v_{te}=\sqrt{{v_{t}}^{2}+{v_{e}}^{2}}, \end{array}$$

(42) $$\begin{array}{c} \theta_{fd}=atan2\left(v_{f},\;v_{d}\right),\;\;\theta_{fe}=atan2\left(v_{f},\;v_{e}\right),\;\;\theta_{td}=atan2\left(v_{t},\;v_{d}\right),\;\;\theta_{te}=atan2\left(v_{t},\;v_{e}\right). \end{array}$$

### 2.6. Planar Body Control

The linear and angular velocities model used for the robot body can be simplified using a coordinate transformation model from the inertial system velocities to the robot body, as presented in Equation (43). Due to the presence of sines and cosines, this equation has nonlinearities. To linearize the equation, a feedback linearization method is used. Using the actuation vector ${\left[v_{rx},v_{ry},w_{r}\right]}^{T}$, it is possible to linearize the system using Equation (44):

(43) $$\begin{array}{c} \left[\begin{array}{c} v_{x}\\ v_{y}\\ \omega \end{array}\right]=\left[\begin{array}{ccc} cos(\theta ) & -sin(\theta ) & 0\\ sin(\theta ) & \;\;\;\;cos(\theta ) & 0\\ 0 & 0 & 1 \end{array}\right]\left[\begin{array}{c} v_{rx}\\ v_{ry}\\ \omega_{r} \end{array}\right], \end{array}$$

(44) $$\begin{array}{c} \left[\begin{array}{c} v_{rx}\\ v_{ry}\\ \omega_{r} \end{array}\right]=\left[\begin{array}{ccc} cos(\theta ) & sin(\theta ) & 0\\ -sin(\theta ) & \;\;\;\;cos(\theta ) & 0\\ 0 & 0 & 1 \end{array}\right]\left[\begin{array}{c} u_{ax}\\ u_{ay}\\ u_{aw} \end{array}\right]. \end{array}$$

The vector ${\left[u_{ax},u_{ay},u_{aw}\right]}^{T}$ is the auxiliary control action for linear and angular velocities. For this auxiliary control, a proportional control with feed-forward was used to follow a curve. For the orientation control, on the other hand, only proportional control was adopted, following the methodology proposed in [52]. The auxiliary control is represented in Equation (45). The vector ${\left[v_{xd},v_{yd},0\right]}^{T}$ corresponds to the feed-forward component of the controller, while the terms $k_{x}$, $k_{y}$ and $k_{\theta }$ are the proportional gains of the controller. The error vector for in-plane position and orientation is composed of the terms $p_{xd}-p_{x}$, $p_{yd}-p_{y}$ and $\theta_{d}-\theta$. During motion, the robot remains aligned with the direction of the curve. To orient the robot with the direction of motion, the term $\theta_{d}$ is calculated using the *atan*2 function of the components $u_{ax}$ and $u_{ay}$, as proposed in [52], similar to the feed-forward action for orientation, as presented in Equation (46):

(45) $$\begin{array}{c} \left[\begin{array}{c} u_{ax}\\ u_{ay}\\ u_{aw} \end{array}\right]=\left[\begin{array}{c} v_{xd}\\ v_{yd}\\ 0 \end{array}\right]-\left[\begin{array}{ccc} k_{x} & 0 & 0\\ 0 & k_{y} & 0\\ 0 & 0 & k_{\theta } \end{array}\right]\left[\begin{array}{c} p_{xd}-p_{x}\\ p_{yd}-p_{y}\\ \theta_{d}-\theta \end{array}\right], \end{array}$$

(46) $$\begin{array}{c} \theta_{d}=arctan2\left(u_{ax},\;u_{ay}\right). \end{array}$$

Linearizing Equation (43) with Equation (44) and replacing the auxiliary controls from Equation (45) with linearized Equation (43), we find Equation (47). This equation represents the error dynamics, where the position error and angular velocity tend to zero over time:

(47) $$\begin{array}{c} \left[\begin{array}{c} {\dot{e}}_{x}\\ {\dot{e}}_{y}\\ \omega \end{array}\right]=-\left[\begin{array}{ccc} k_{x} & 0 & 0\\ 0 & ky & 0\\ 0 & 0 & k_{\theta } \end{array}\right]\;\left[\begin{array}{c} e_{x}\\ e_{y}\\ e_{\theta } \end{array}\right]. \end{array}$$

This control methodology allows us to obtain the desired linear and angular velocities for the robot body using Equation (44). Based on these velocities and Equations (38)–(42) the leg velocities are calculated, which are then sent as a reference to the step generation control.

### 2.7. General Description of Control Strategy

To implement our control strategy, we use the Locosim robotics framework [36], which consists of a ROS Noetic node written in Python v3.8, for high-level planning and control, interfacing with a C++ ROS Noetic control node for low-level control to a Gazebo v11 simulator or physical hardware. We used the Unitree Go1 quadruped robot platform (Unitree, Hangzhou, China) for both simulations and real hardware experiments. The Unitree Go1 robot has twelve motors with a maximum torque of 23.7 Nm and a maximum speed of 30 rad/s. The Unitree Go1 robot description and hardware interface is integrated into the Locosim framework.

**Figure 7:**
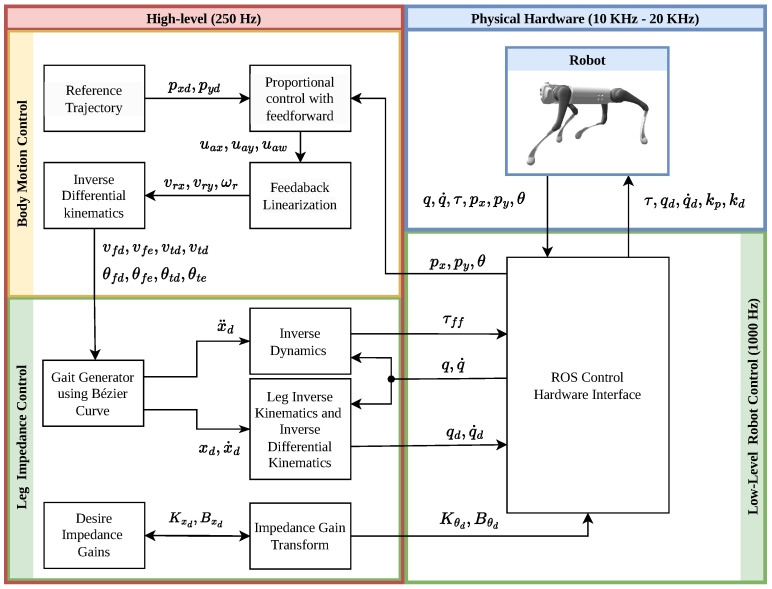
Diagram of the control strategy. The high-level node implemented in (red box), which includes the body control (yellow box) and leg control (green box inside red box). The low-level node implemented in C++ (green box) and the simulated or physical hardware (blue box).

For our proposed body and leg control strategy shown in Figure 7, we developed a custom high-level Python ROS Noetic node (red box), a low-level C++ ROS Noetic node (green box) and a hardware interface based on Locosim QuadrupedController, ros_impedance_controller and go1_hardware_interface, respectively.

The high-level node runs at 250 Hz. It uses Pinocchio [37] to calculate the kinematics and dynamics of the robot. The body control (yellow box inside the red box) receives the body reference trajectory ($p_{xd},p_{yd}$) and computes the proportional control with the feed-forward of Equation (45), the feedback kinematic linearization of Equation (44), and  inverse differential kinematics of Equations (38) and (40). This results in the velocity of Equation (41) and the velocity direction of Equation (42) of each leg.

The high-level leg control (green box inside red box) receives the velocity and direction of each leg and computes the gait generator using Bézier curves, as detailed in Section 2.4. We consider the desired inertia as $M_{x_{d}}={J(q)}^{-T}M\left(q\right){J(q)}^{-1}$ to maintain the inertia of the system. Substituting this into Equations (10) and (11), we obtain the feed-forward torque for each leg:

(48) $$\tau_{ff}=M\left(q\right){J(q)}^{-1}\left({\ddot{x}}_{d}-\dot{J}\left(q,\dot{q}\right)\dot{q}\right)+C\left(q,\dot{q}\right)\;\dot{q}+g\left(q\right),$$

and the feedback torque:

(49) $$\tau_{fb}={J(q)}^{T}\left(K_{x_{d}}\left(x_{d}-x\right)+B_{x_{d}}\left({\dot{x}}_{d}-\dot{x}\right)\right).$$

It is important to note that with this consideration, the impedance control is equivalent to a position-proportional-derivative feedback controller, eliminating the need to estimate external forces.

To maintain stability setting high impedance gains, it is required to send motor commands to the robot directly. The motor command operates in the frequency range of 10 KHz to 20 KHz. Each Go1 motor command accepts desired torque, joint angles, joint angular velocities, proportional, and derivative gains. To send the motor command, we convert the feedback torque from task-space to joint-space. This involves a transformation to determine the desired stiffness gain and the desired joint angle using the force–torque relationship $\tau =J^{T}f$ and the inverse kinematics of the leg. Furthermore, we convert the desired damping and foot velocities to the desired joint angular velocities using differential inverse kinematics. This conversion produces the feedback torque in joint-space, represented as:

(50) $$\tau_{fb}=K_{\theta_{d}}\left(q_{d}-q\right)+B_{\theta_{d}}\left({\dot{q}}_{d}-\dot{q}\right),$$

where $K_{\theta_{d}}={J(q)}^{T}K_{x_{d}}J(q)$, $B_{\theta_{d}}={J(q)}^{T}B_{x_{d}}J(q)$, $q_{d}$ derives from the inverse kinematics of $x_{d}$, $\dot{q_{d}}=J{\left(q\right)}^{-1}{\dot{x}}_{d}$, *q* and $\dot{q}$ are the actual joint angle and angular velocity, respectively.

The low-level node (green box) operates at a frequency of 1000 Hz. It receives the high-level control command and sends the Go1 motor command joints’ torques ($\tau_{ff}$), the desired joints’ angles ($q_{d}$), the desired joints’ angular velocities (${\dot{q}}_{d}$), proportional gains ($k_{p}$) and derivatives gains ($k_{d}$). Due to the constraint of the motor command of accepting only one proportional gain and one derivative gain for each joint, we take the diagonal terms of the desired leg stiffness ($K_{\theta_{d}}$) and the desired leg damping ($B_{\theta_{d}}$), as they are the most significant terms. These terms are sent as the proportional gain and the derivative gain for each joint motor.

## 3. Results

The following sections present the results of simulations and tests performed on the Go1 robot. The first subsection presents the simulations of the proposed control conducted on the Gazebo v11 using the Locosim framework, and the second subsection presents the tests carried out on Unitree’s Go1 robot. Videos of simulations and tests performed on hardware are available online: https://github.com/GabrielDGP/Sensors_quadruped_robot_control.git (accessed on 3 May 2024).

### 3.1. Simulations

First, a simulation was conducted to verify the leg control in following the Bézier curves with the legs. Then, the robot was tested on the ground with the same configuration. Finally, the body control was tested using a lemniscate path. Gains $M_{x_{d}}$, $B_{x_{d}}$, and $K_{x_{d}}$ were adjusted to obtain the mass-spring-damper effect on the legs while following the trajectory of the Bézier curve. The gains found were $M_{x_{d}}=J^{-T}M\left(q\right)J^{-1}$, $B_{x_{d}}=44\;I$, $K_{x_{d}}=1000\;I$. Regarding body control, proportional gains were adjusted to follow the reference trajectory, with $k_{x}=k_{y}=0.55$ and $k_{\theta }=3.5$ being the values that represent the best performance.

#### 3.1.1. Leg Control following Bézier Curves

**Figure 8:**
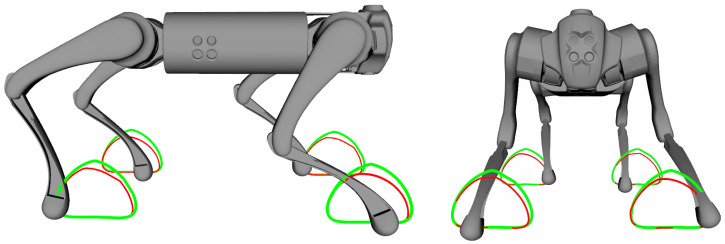
Frontal (**left**) and lateral (**right**) movements following Bézier curves. Trajectory of the Bézier curve B(u) (green line) and final position of the robot feet (red line).

Initial simulations aimed to verify whether the proposed control for robot legs could follow the Bézier curves used as a reference trajectory. The robot was suspended in the simulation to test the movement of its legs in the front and lateral directions. Figure 8 illustrates the results of simulations by comparing the trajectory of the Bézier curve B(u) (green line) with the final position of the robot feet (red line) with a step frequency of 1.0 Hz.

**Figure 9:**
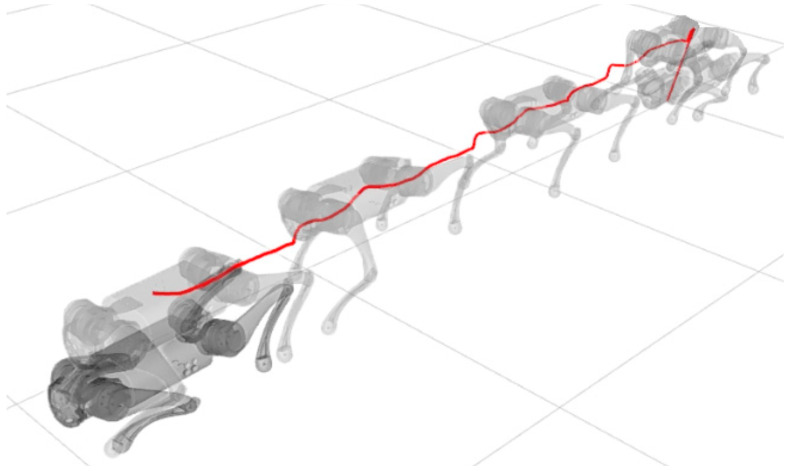
Body trajectory static walk.

**Figure 10:**
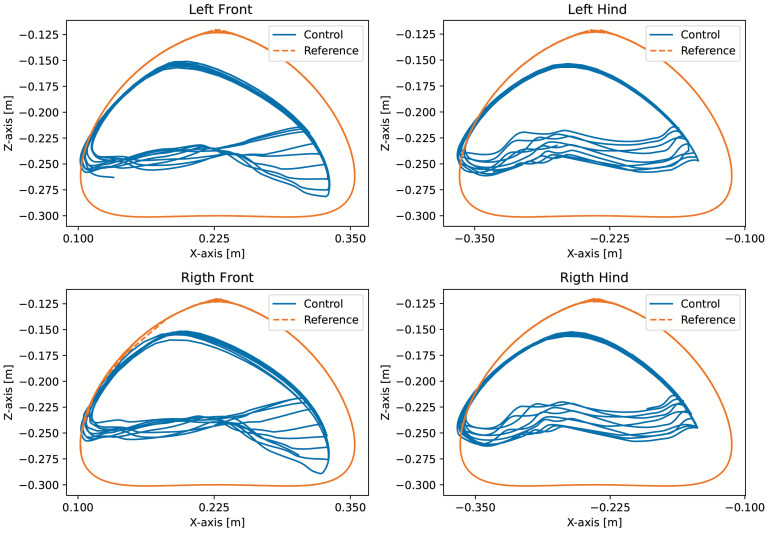
Trajectory of feet static walk.

**Figure 11:**
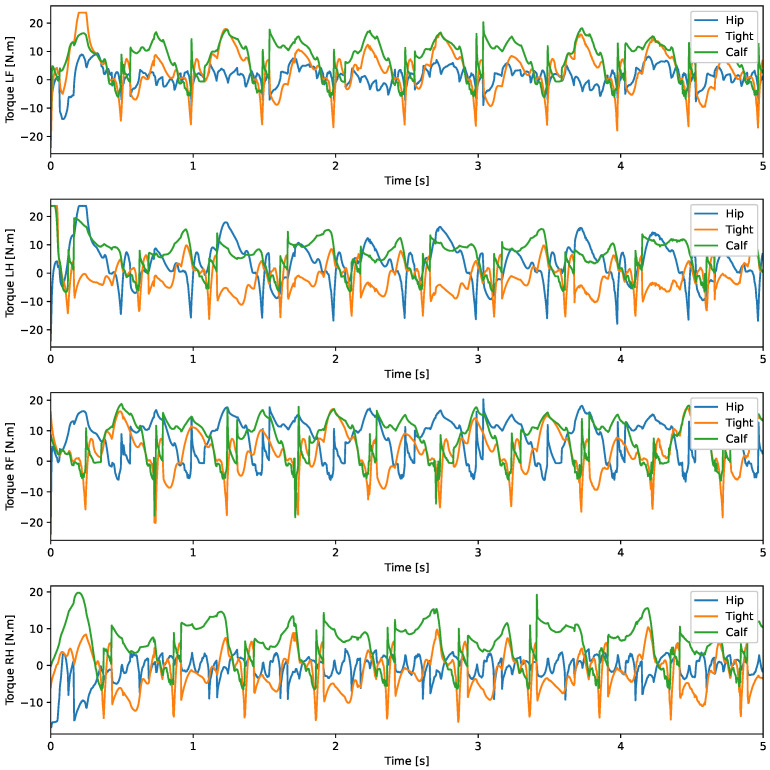
Joint torque static walk.

**Figure 12:**
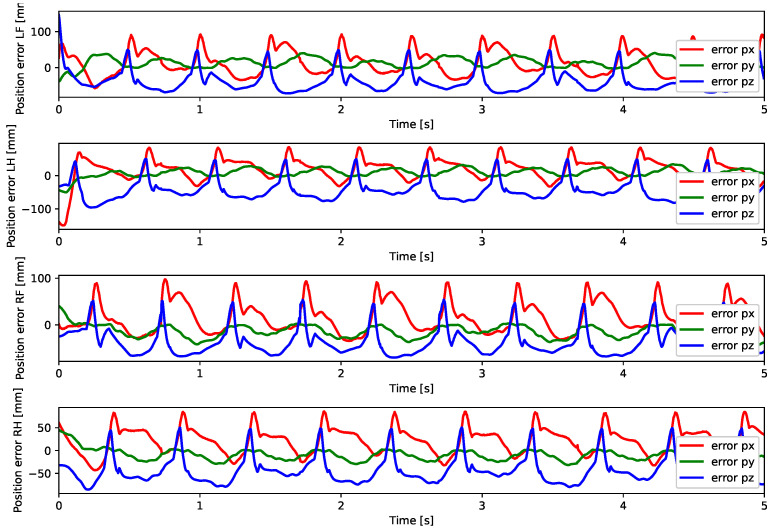
Foot position error static walk.

**Figure 13:**
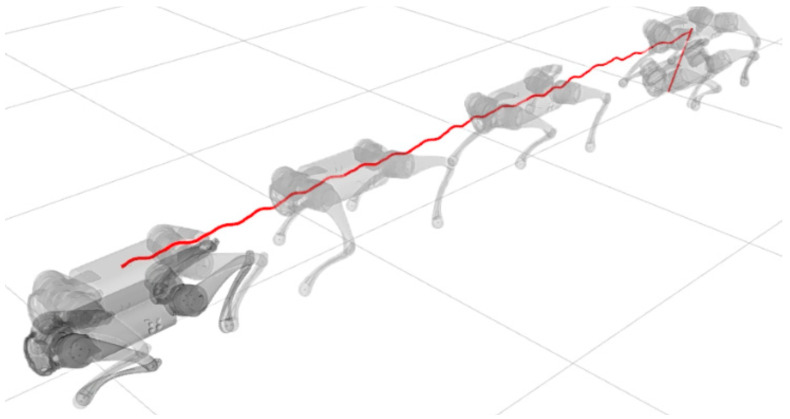
Body trajectory trot.

**Figure 14:**
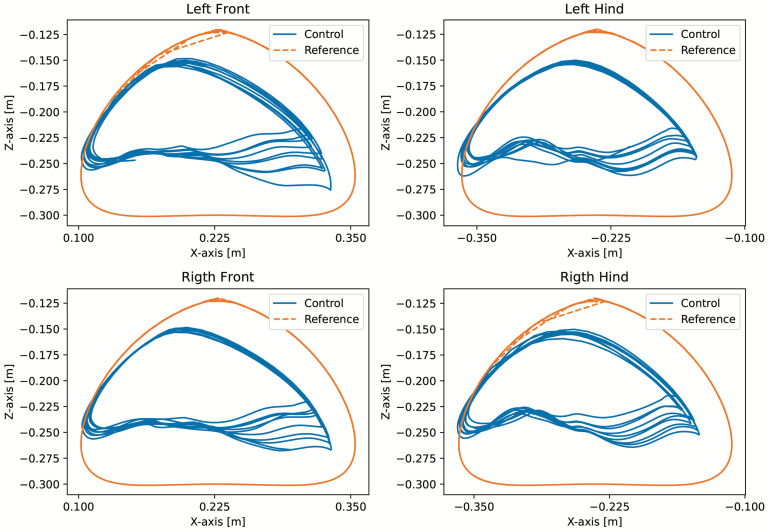
Trajectory of feet trot.

**Figure 15:**
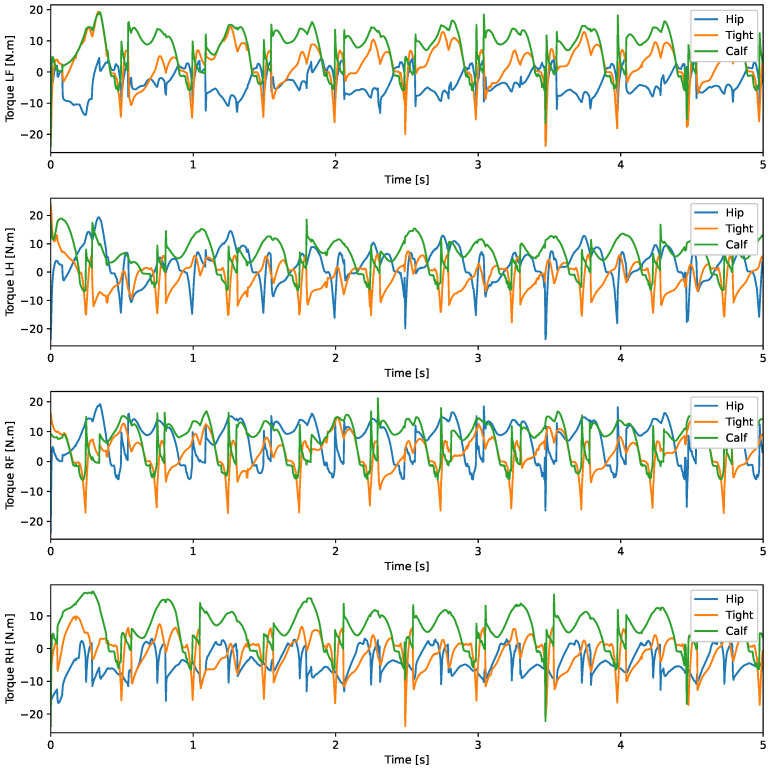
Joint torque trot.

**Figure 16:**
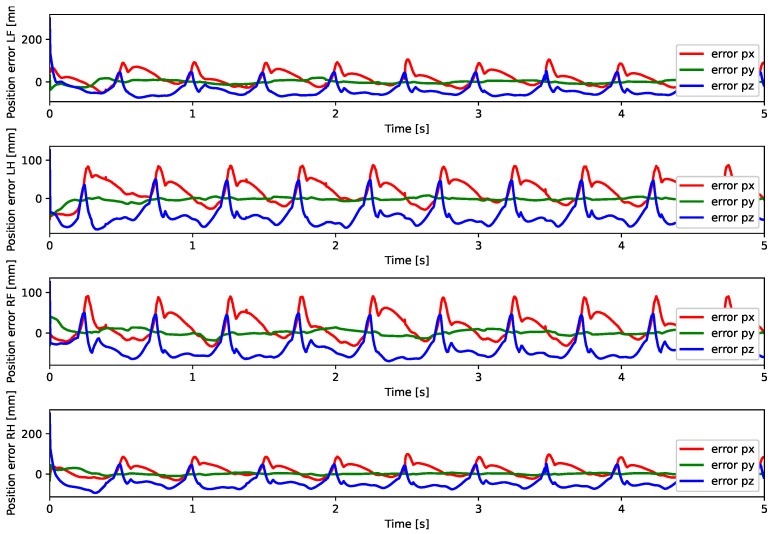
Foot position error trot.

Subsequent simulations evaluated the synchronized control of the legs during locomotion of the robot resting on the ground. We set the forward speed of 1 m/s, and for walking patterns, we chose the trot gait. The trajectory of the body, trajectory of the feet, torques and error dynamics of static walk and trot gait can be seen in Figure 9, Figure 10, Figure 11, Figure 12, Figure 13, Figure 14, Figure 15 and Figure 16.

#### 3.1.2. Body Control following Lemniscate Curve

**Figure 17:**
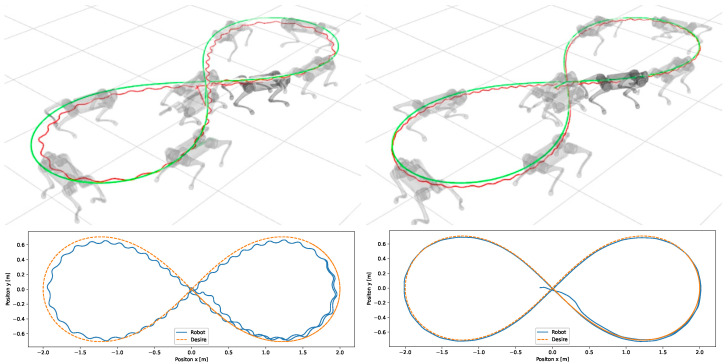
Simulations of the control of the robot body and legs static walk gait (**left**) and trot gait (**right**); reference in green and robot in red.

Lastly, we simulate the control following a Bernoulli-lemniscate curve with a length of 2 m and an angular velocity of $15^{\circ }$/s as a reference trajectory. The robot’s ability to execute left and right turns was tested using this curve for static walk and trot gait (Figure 17).

### 3.2. Physical Robot Go1

The hardware experiments were carried out using the locosim framework. The first test was with the robot suspended in the air to assess whether the legs followed the trajectory of the Bézier curves for the steps. The second test involved the robot on the ground, with a speed of 1 m/s to the controller, using the trot gait pattern. The third test was carried out only with the body control of the robot passing the velocities in the x, y and angular velocity in z directions. These speeds are sent to Unitree’s motion control and were generated by the body controller to follow the trajectory of Bernoulli’s lemniscate. The gains $M_{x_{d}}$, $B_{x_{d}}$, and $K_{x_{d}}$ were adjusted with the same gains used in simulation to obtain the mass-spring-damper effect on the legs while following the trajectory of the Bézier curve. The gains used were $M_{x_{d}}=J^{-T}M\left(q\right)J^{-1}$, $B_{x_{d}}=44\;I$, and $K_{x_{d}}=1000\;I$. Regarding body control, proportional gains were adjusted to follow the reference trajectory, with $k_{x}=k_{y}=0.55$ and $k_{\theta }=3.5$ being the values that represent the best performance.

#### 3.2.1. Leg Control following Bézier Curves in Hardware

**Figure 18:**
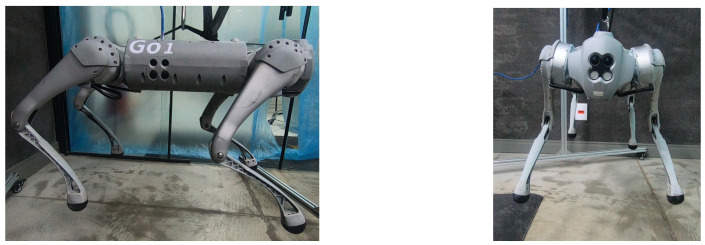
Tests with the physical robot suspended in the air.

**Figure 19:**
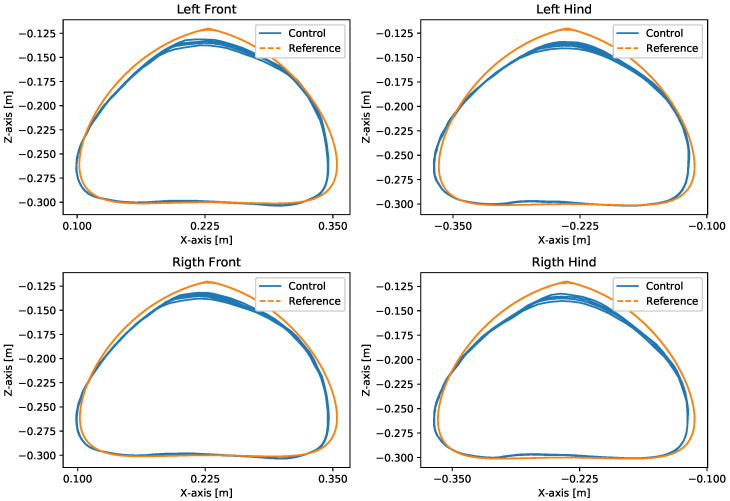
Movement in the forward direction on the suspended real robot.

**Figure 20:**
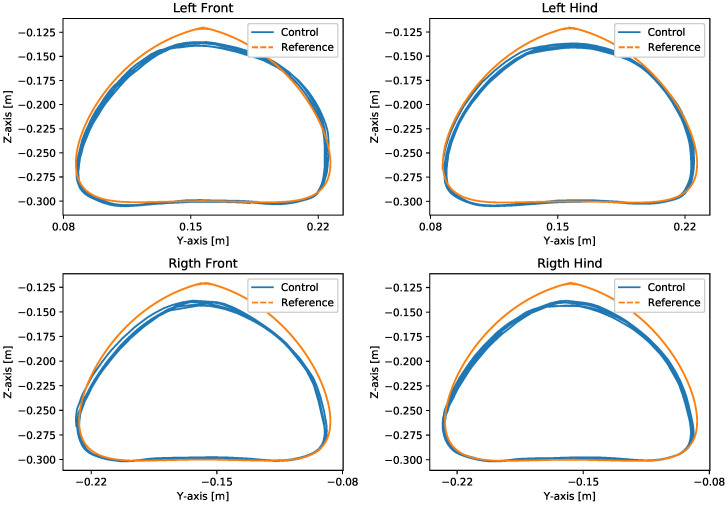
Movement in the lateral direction on the suspended real robot.

With the robot suspended in the air, Figure 18, the trajectories of the Bézier curve for frontal and lateral movement were provided with a step frequency of one step per second to check whether the physical robot could follow this trajectory. The result of the path taken by the physical robot’s feet and the torques of motors for this movement can be seen in Figure 19 and Figure 20.

**Figure 21:**
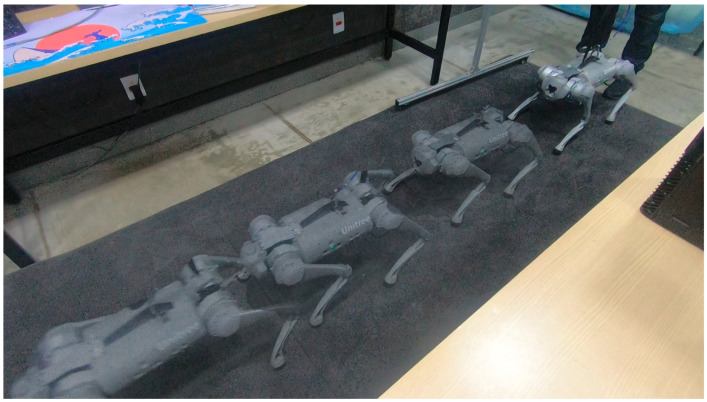
Robot on ground performing trot gait.

**Figure 22:**
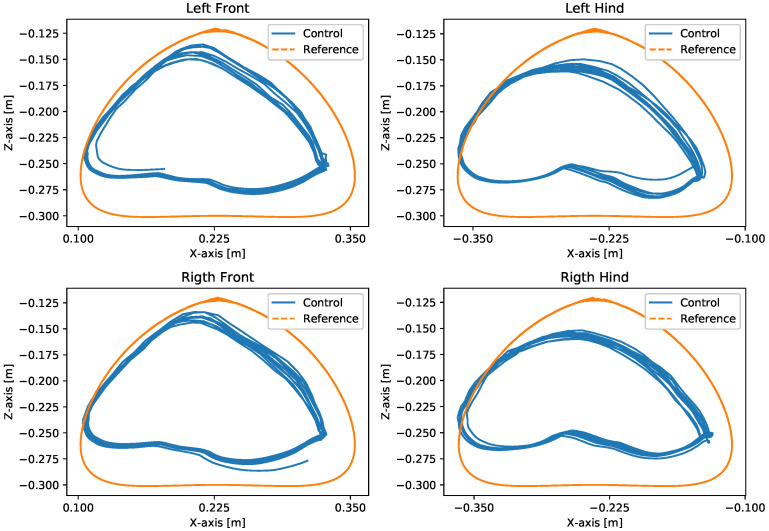
Trajectory of feet trot physical robot.

**Figure 23:**
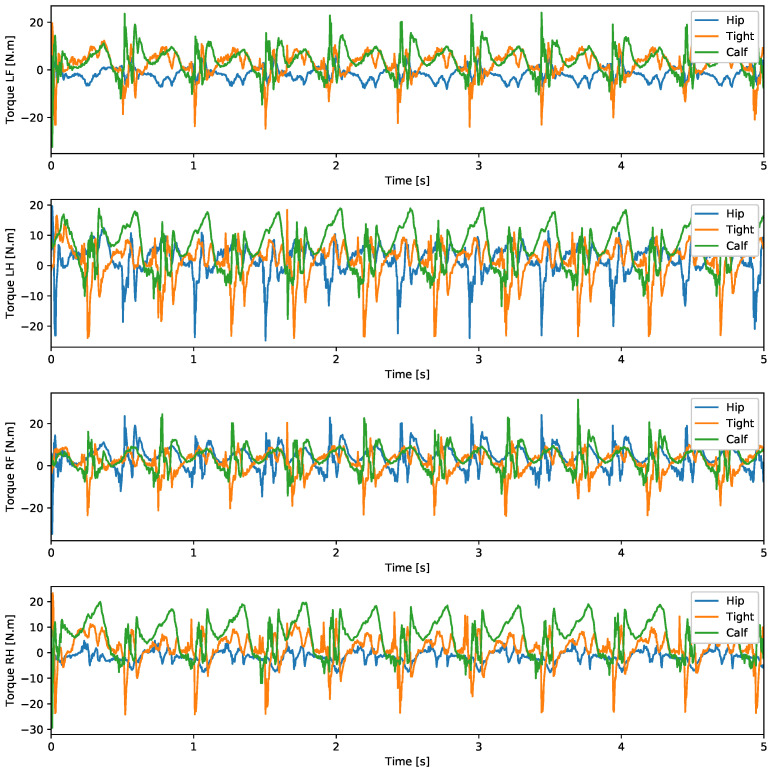
Joint torque trot physical robot.

**Figure 24:**
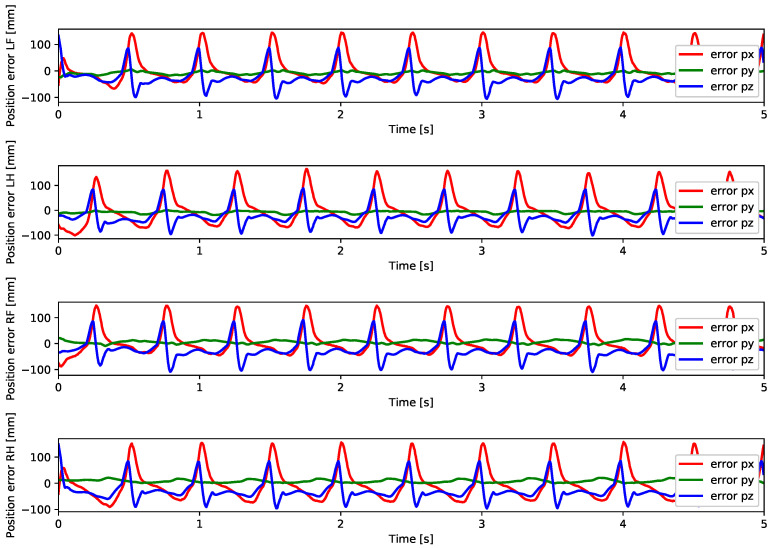
Foot position error trot physical robot.

A test was also carried out with the robot on the ground to check whether the proposed controller can make the real robot stand up and walk. The robot was able to get up and walk with the trot gait pattern, as shown in Figure 21; the foot trajectory, motor torques and foot position errors of that walk test are shown in Figure 22, Figure 23 and Figure 24.

#### 3.2.2. Planar Body Control

**Figure 25:**
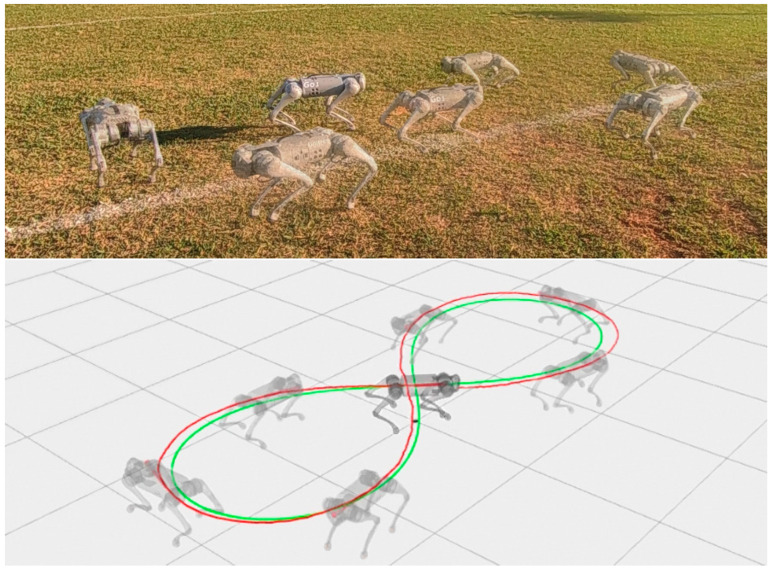
Real robot following lemniscate curve with proposed body control and Unitree motion control. Reference trajectory in green and the robot’s executed trajectory in red.

It was not possible to test the control of the body in conjunction with the control of the legs on the real robot, as the low-level communication of the Go1 robot does not permit the use of its odometry system, and this article does not cover the problem of odometry for mobile robots with legs; the only option for motion control in high-level communication is the one provided by Unitree. In this way, the body control test was performed using the high-level communication that has access to the odometry and motion control of the Go1 robot developed by Unitree. The test was carried out only with the body control of the robot passing the velocities in the x, y and angular velocity in z directions. These speeds were sent to Unitree’s motion control through a cmd_vel command and were generated by the body controller to follow the trajectory of Bernoulli’s lemniscate. The results of this test can be seen in Figure 25.

## 4. Discussion

This section discusses the results of experimenting with the proposed control strategy in simulation and on a physical robot. The first subsection presents the results of the simulation and the improvements that enabled a simulation real-time factor of one. The second subsection presents the results of the tests conducted on the physical robot.

### 4.1. Simulation Discussion

During the first simulation, it was observed that the proposed leg control was able to follow the reference trajectories of the Bézier curve in both frontal and lateral movement (Figure 8), with minimal error between the reference curve and the actual curve traced by the robot’s feet. The reference Bézier curve has a duty factor of 0.8 and a step height of 100 mm. The error can be reduced by increasing the desired stiffness $K_{x_{d}}$ of the leg. However, this may result in reduced compliance of the leg, making it unable to effectively absorb reaction forces and potentially leading to instability or falling.

In the second simulation, we observed that the robot was able to move with static walking and trotting gait patterns. The trajectory of the feet does not fully follow the reference of the Bezier curve, as shown in Figure 10 and Figure 14, as is expected from an impedance control. The gains of this control have been adjusted so that the feet absorb the impact of the steps and still follow part of the movement provided by the trajectory planning even at high step frequencies. The torques sent to the robot’s joints responded to external forces applied to the foot tip (Figure 11 and Figure 15). Additionally, we analyzed the foot support and swing cycles in the foot position error dynamics (Figure 12 and Figure 16). The error deviated from zero when forces were applied to the feet, as expected with impedance control. The torque values recorded during these actions remained below the maximum torque limit of 23.7 Nm for the Go1 motor. When comparing the static walking and trotting gait patterns in relation to the robot’s body movement, it is noticeable that in static walking the body has greater lateral movements, while in the trotting locomotion pattern, the robot has a greater movement of the body up and down, especially when locomoting with a step frequency below 1 step per second and a high step height. During the simulations, the height of the base was kept constant at 0.3 m, which is a good value because the legs are maintained within a safe distance from the kinematic limits and still allow for some maneuverability. As for the feet trajectories, the reference step height used in the simulations was defined as 0.175 m. On flat terrain, this step height is sufficient for the feet not to touch the ground and still enables the robot to overcome some variations in the terrain height. In more unstructured terrains, a higher step height can be used so that the impedance control can raise the feet above the height of the obstacles but with a low stiffness to comply with the terrain variations without losing stability. However, the use of a step height too high can cause the base of the robot to wobble and tip over. For more difficult terrains, it might be necessary to employ a more sophisticated step-planning approach that takes into account a terrain elevation map obtained from sensor data, such as LIDAR or depth cameras. However, step planning with elevation maps is not covered in this article.

The third set of simulations demonstrated that the body position control was capable of guiding the robot’s reference coordinate system to track the trajectory of the lemniscate during both static walking and trotting gaits. The leg control gains were configured as $M_{x_{d}}=J^{-T}M\left(q\right)J^{-1}$, $B_{x_{d}}=44\;I$, and $K_{x_{d}}=1000\;I$ desired inertia, damping, and stiffness, respectively. These gain settings allowed the leg to absorb impact forces during walking. This allowed the robot to better follow the trajectory of the lemniscate curve along with the body control, although the trajectories of the steps of the Bézier curve were not closely followed. As the robot approached the reference trajectory, it steadily minimized position errors over time (Figure 17a,b). This simulation also showed that the static walking gait pattern had a greater lateral movement of the body, with a sinusoidal behavior when following the trajectory of the lemniscata. The trot gait pattern maintained the trajectory of the body with few oscillations due to the frequency of two steps per second, with a greater oscillation in the stretches in the center of the lemniscata which have a lower speed compared to the trajectory of the curves.

The results were similar to those obtained in the previous work [34], with the difference being that the simulations were carried out in the Gazebo v11 simulator with a real-time factor of one. The C++ ROS Noetic node for impedance control enabled faster calculations, resulting in a faster simulation and allowing for lower controller gains compared to the previous work. Another approach that allowed the control to be easier to implement was setting the desired inertia $M_{x_{d}}=J^{-T}M\left(q\right)J^{-1}$. In this way, the control equations are simplified, and there is no need for force feedback from the feet, which makes it easier to implement this control on a real robot, and we also simplified the equations for the equivalent of a position PD feedback controller that does not need to multiply the inertia matrix $M\left(q\right)J{\left(q\right)}^{-1}$, just $J{\left(q\right)}^{T}$ [53].

### 4.2. Physical Robot Experiments Discussion

The first experiment conducted using the physical robot aimed to assess its capability to track a Bézier curve for the robot’s stepping motion. The gains used were the same as in the simulation. As shown in Figure 19 and Figure 20, the robot follows the Bézier curve with low error when using a low speed and frequency of steps.

In tests with the robot on the ground, it was able to get up and move with the trot locomotion pattern with the front speed command of 1 m/s. The trot locomotion pattern was chosen for this test with the real robot because it was the locomotion pattern that had the best performance in the simulations, with few lateral body movements compared to the static walking pattern. The foot trajectory, motor torques and foot position error were similar to those in the simulation, as shown in Figure 21, Figure 22, Figure 23 and Figure 24, with the impedance control allowing the feet to absorb the impact of the steps while following the foot movement trajectory provided by the Bézier curves. This was only possible by sending the desired joint’s feed-forward torque, angle, angular velocity, and impedance gains directly to the Go1 joint motors. This was accomplished by sending the desired joint’s feed-forward torque, angle, angular velocity, and impedance gains directly to the Go1 joint motors. We only sent the diagonal terms of the desired stiffness and damping because the joint motor command only accepts one proportional gain and one derivative gain per joint. While this approach is not ideal due to the non-equivalence of gains in joint space and task space, focusing on the diagonal terms proved effective. These terms are more significant compared to the off-diagonal terms and successfully controlled the robot both in simulation and on physical hardware.

The body control test provided the robot’s $V_{rx}$, $V_{ry}$ and $W_{r}$ velocities, which were sent to Unitree’s Go1 high-level controller through a cmd_vel command. When receiving the linear and angular velocity commands, the robot successfully followed the Bernoli lemniscate trajectory specified in the proposed body control framework, aligning its orientation with the direction of movement [52]. Compared to the simulation results, the robot’s body exhibited fewer oscillations of its center of mass because Unitree’s motion control uses the dynamics of the robot’s whole body and trot gait pattern.

## 5. Conclusions

This paper presents a planar motion control strategy for a quadruped robot, employing Bézier curves to generate two distinct gait patterns for the robot’s feet. Our control implementation integrates body control with leg control, incorporating impedance control based on the dynamic model of each leg. The gait patterns of static walk and trot are realized using 6th-degree Bézier curves. Body control is achieved through a planar kinematic model. The control strategy was implemented using the Locosim framework in conjunction with ROS, and it was validated using Unitree’s Go1 robot through simulations in Gazebo v11, as well as physical hardware.

In simulation, the impedance control of the legs coupled with body control worked as expected, enabling the robot to execute trot and static walk gaits while following the Bernoulli lemniscate curve. The impedance control applied to the physical robot allowed the robot to move successfully in a trotting pattern similar to the simulations carried out. The efficacy of Bézier curves as a mathematical tool for generating adaptable trajectories was demonstrated, including in applications involving the physical robot. The proposed planar body control successfully tracked a trajectory based on a Bernoulli lemniscate curve, both in simulation and on the physical robot, aligning the robot with the direction of movement specified by the trajectory.

Future work includes the development of a gait generator capable of dynamically modifying its parameters to enable the robot to move at higher speeds or traverse irregular terrain. This could be achieved through the use of cameras or laser sensors to map the ground and adapt the gait accordingly. Another important aspect of moving on uneven terrain is controlling the body’s orientation in multiple directions. In this context, further work could involve developing a kinematic model for body orientations in the x and y directions, along with a control mechanism for these orientations. Furthermore, how to include off-diagonal terms of the stiffness and damping matrix in the joint motor command of the robot can be investigated. Finally, an additional future work includes modifying the proposed control strategy by exploring alternative kinematic or dynamic models that consider movement in three-dimensional space and take into account disturbances caused by external forces acting on the robot’s body.

## Data Availability

Data are contained within the article.
